# Vitamin K Properties in Stroke and Alzheimer’s Disease: A Janus Bifrons in Protection and Prevention

**DOI:** 10.3390/molecules30051027

**Published:** 2025-02-24

**Authors:** Lorenzo Grimaldi, Rosaria A. Cavallaro, Domenico De Angelis, Andrea Fuso, Giulia Sancesario

**Affiliations:** 1 IRCCS Santa Lucia Foundation, European Center for Brain Research, 00179 Rome, Italy; 2 Department of Surgery, Sapienza University of Rome, 00161 Rome, Italy; 3 Department of Experimental Medicine, Sapienza University of Rome, 00161 Rome, Italy; 4 Center for Research in Neurobiology, Sapienza University of Rome, 00161 Rome, Italy

**Keywords:** vitamin K, biological activity, stroke, Alzheimer’s disease, coagulation, vascular calcification, amyloid B, neurodegeneration

## Abstract

Vitamin K is essential for many physiological processes, including coagulation, bone metabolism, tissue calcification, and antioxidant activity. Vitamin K vitamers are represented by lipophilic compounds with similar chemical structure (i.e., phylloquinone (vitamin K1) and menaquinone (vitamin K2)). Vitamin K deficiency can affect coagulation and vascular calcification, increasing the risk of hemorrhages, atherosclerosis, cerebrovascular diseases, and neurodegeneration. Recently, several studies have hypothesized a possible dual role of vitamin K vitamers in benefiting both vascular and cerebral health, e.g., by sphingolipids biosynthesis or ferroptosis inhibition. The aim of this narrative review is to deepen the understanding of biological activities of vitamin K and its possible dual protective/preventive actions in neurovascular and degenerative conditions, e.g., stroke and dementia. Given the difficulties related to hemorrhagic risk entailed in the prevention of strokes, the function of vitamin K antagonists is also investigated. Finally, we track the development of a clinical concept for a future preventive strategy and innovative use of vitamin K as a supplement to counteract neurovascular and pathological processes, focusing in particular on stroke and dementia.

## 1. Introduction

Vitamin K (Vit K) is essential for many physiological processes, including coagulation, bone metabolism, tissue calcification, and antioxidant activity. Vit K is a cofactor in the post-translational activation of both procoagulant and anticoagulant factors as well as proteins related to bone formation inflammation and apoptosis [1,2,3,4,5,6,7,8]. Vit K, so called from the first letter of the German word ‘‘Koagulation’’, is a group of lipid-soluble compounds first discovered by the biochemist H.C.P. Dam in 1929 as a key factor in blood clotting by observing coagulation disorders with severe bleeding in chicken fed with a low-fat diet [9]. For the importance of these studies, he was awarded the Nobel Prize in 1943 together with E. A. Doisy, who discovered the Vit K chemical structure [10]. In the last decades, growing evidence has shown that Vit K may have many functions beyond coagulation, including cardiovascular, brain, and bone health, immune response, and inflammation regulation, other than antioxidant activity and potential anti-cancer and amyloidogenic effects [11,12,13,14,15]. In recent years, possible epigenetic mechanisms associated with Vit K have also been suggested [16,17,18]. This extends the interest beyond coagulation and cardiovascular diseases, focusing on its involvement in the pathogenesis of complex chronic diseases, such as neurological ones. In fact, vascular disorders often coexist in dementia and are strongly associated with increased risk of both stroke and dementia.

Vit K acts as a cofactor in the gamma-carboxylation of the glutamic acid residues of hepatic and extrahepatic Vit K-dependent proteins (VKDPs), resulting in their activation [11,19,20]. VKDPs include protein related to the blood coagulation process, bone development, and cardiovascular and brain health [5,11,21,22]. Further, Vit K is involved in the biosynthesis of sphingolipids, an important component in the central nervous system [23,24,25], and active in inhibition of ferroptosis, a non-apoptotic form of cell death characterized by iron-dependent lipid peroxidation (UdMO1).

In this scenario, to evaluate whether Vit K may be employed as a protective or preventive supplement is of paramount importance due to the potential benefit for the public health and management of patients with high cost and incidence pathologies. The aim of this narrative review is to deepen the understanding of biological activities of Vit K in physiological conditions and its possible dual protective/preventive actions in stroke and dementia; first, we describe structures of Vit K forms, how they can be obtained by diet, and specific bioavailability; second, we discuss the wide biological properties of Vit K and related Vit K-dependent proteins (VKDPs). Third, we provide a state of the art on effects of Vit K deficiency on different physiological pathways, e.g., coagulation or calcium homeostasis, and track the development of a clinical concept for a future innovative use of Vit K as a supplement to counteract neurovascular and pathological processes, focusing in particular on stroke and dementia.

## 2. Vitamin K

### 2.1. Structure

**Figure 1:**
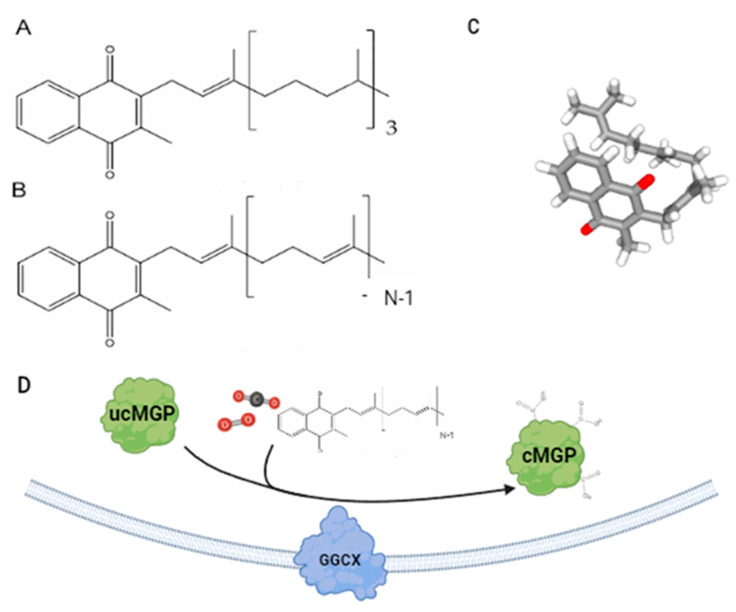
Chemical structure of Vit K vitamers: phylloquinone or Vit K1 (**A**) and menaquinone-n (MKn) and Vit K2 (**B**) with menaquinone-3 3D structure (Bond color scheme: gray, carbon-carbon bonds; white, carbon-hydrogen; red, carbon-oxygen) (**C**) (National Center for Biotechnology Information (2025). PubChem Compound Summary for CID 5280483). (**D**) Carboxylation catalysis of Matrix Gla Protein. Integral membrane protein gamma-glutamyl carboxylase (GGCX) uses oxygen, carbon dioxide, and the reduced form of Vit K as cofactors to catalyze the subtraction of the gamma-hydrogen of glutamate residues and the addition of a carboxyl group. Chemical energy for the carboxylation is extracted from the oxidation of Vit K to Vit K epoxide [34]. (This image was created with BioRender.com (https://www.biorender.com/ accessed on 10 January 2025).

The term Vit K refers to a family of structurally similar compounds, sharing a common ring core, the 2-methyl-1,4-naphthoquinone, called menadione. Natural forms of Vit K consist in two vitamers, the phylloquinone, or Vit K1, and Vit K2, also known as menaquinones, a group of molecules different in terms of the characteristic of the side chain at the C3 of the quinone ring (Figure 1). Vit K1 contains a long phytyl side chain with four prenyl units, while Vit K2 menaquinones contain a poli-isoprenoid side chain, variable in the number “*n*” of isoprene units, that identify the menaquinone as MKn, with “*n*” between 4 and 13 [8,25,26,27,28]. They are short-chain menaquinones, like MK4, and long-chain menaquinones, like MK7. Due to the characteristic of the isoprenic side chain, menaquinones can exist as cis and trans isomers, [26,27,29]; cis phylloquinone has only 1% of the biological activity in respect to the *trans* form [30,31]. For this reason, the quality and safety of Vit K nutritional supplements must be studied, since they may contain significant amounts of isomers with no or almost unknown biological activity. Menadione, also known as Vit K3, is a synthetic form of Vit K, without any substituent at C3, and despite its reported toxic effects, it is often used as a Vit K source in animal husbandry [16,21,32]. Other synthetic forms of Vit K are Vit K4 (menadiol acetate), K5, K6, and K7 [12]. These synthetic vitamers of Vit K are being studied for potential applications. For example, Vit K3 has been used in treating amyloid diseases exhibiting dose-dependent inhibition of fibril formation [33].

### 2.2. Dietary Sources

**Figure 2:**
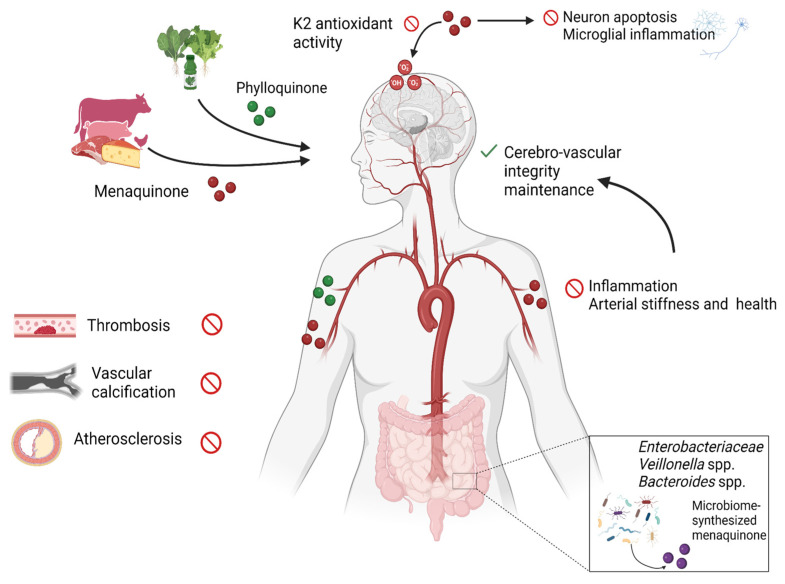
Vit K vitamer food sources and effects on the cerebrovascular system. Dietary sources of menaquinone and phylloquinone are meat and dairy products and vegetables, respectively. Menaquinone can also be synthesized internally by certain genera of intestinal bacteria in the microbiota. Vit K vitamers influence cerebrovascular health through promotion of vascular health, preventing vascular calcification, thrombosis, and atherosclerosis. Reduction in these conditions results in decreased risk of stroke and thus protection of brain function and health. (This image was created with BioRender.com (https://www.biorender.com/ accessed on 10 January 2025).

Vit K natural dietary sources depend on the kind of vitamer: Vit K1, the most abundant form in our diet sources (75–90% of all Vit K), is mainly found in green leafy vegetables, algae, and cyanobacteria, whereas Vit K2 vitamers are produced by bacteria and found in fermented food, dairy, and meat [11,35,36] (Figure 2). Among vegetables, sauerkraut is noteworthy, since it contains both Vit K1 and Vit K2. High contents of Vit K1 are found in collard, cabbage, and spinach but also in fruits like kiwi, avocado, and grape. Plant oils, like soybean and olive oil, are important sources of Vit K1 and, when added to green vegetables, increase Vit K bioavailability thanks to the presence of fats [5,11,12]. Japanese traditional food is particularly rich of Vit K: perilla and edible seaweed like wakame contain a large amount of Vit K1 [20,37], while Natto, an aliment produced through the fermentation of soybeans with Bacillus subtilis subspecies Natto, is the richest source of Vit K2, especially MK7 (10,985 ng/g, about 100 times the MK7 amount in cheese) [26,27,38,39], followed by dairy products. Cheeses have variable amounts of menaquinones, depending both on the duration of fermentation and on the bacterial strain used [40]. Dutch hard cheeses contain higher menaquinone levels than softer cheeses; among European cheeses, Munster has higher content of Vit K 2 (80.1 μg per 100 g) than Roquefort (38.1 μg per 100 g), which conversely has the highest amount of Vit K1 [11]. Vit K1, and especially Vit K2, are also found in meat, but the amount is influenced by the origin of the meat; chicken meat and beef liver contain higher levels of Vit K2 [11,41]. Among other sources of Vit K2, there are eggs and fish, with eels notably having higher content of Vit K2 [5,40].

Vit K2 is produced by some bacterial strains like *Escherichia coli* and *Staphylococcus aureus*; furthermore, bacteria in the human gut microbiome like *Eubacterium lentum*, *Veillonella*, *Enterobacteria*, and *Bacteroides* synthesize longer-chain MKs, but since their bioavailability is very low, the diet represents the main font of Vit K [12,42,43,44]. While long-chain MKs are produced by bacteria, MK4 is synthesized from Vit K1 by a tissue-specific conversion catalyzed by the enzyme UbiA prenyltransferase containing 1 (UBIAD1), occurring in several tissues like kidney, cerebrum, pancreas, and liver [45].

### 2.3. Bioavailability

Although Vit K1 and Vit K2 share the same biological activity, as cofactors in the γ-carboxylation reaction of VKDPs, they differ in terms of pharmacokinetics, bioavailability, absorption rate, and tissue distribution, depending on the structural difference and the length of the side chain. Shurgers and Vermeer [36,46], in a comparative study on the different absorption between Vit K1 and Vit K2 from food, showed that Vit K2 (in particular MK7) is most effectively absorbed, with higher bioavailability. Moreover, long-chained menaquinones like MK7 showed significantly longer circulation half-life (MK7 has a half-life time of about three days with only 1–2 h for K1) [38,47,48], so they are more involved in extrahepatic biological reactions [11,46]. Dietary fats may increase Vit K absorption, e.g., phylloquinone bioavailability from vegetables increases by about three times in the presence of fats, like butter or oil [5]. Notably, MK7 supplementation leads to a lasting significant increase in MK7 serum levels after long-term administration, unlike MK4 and Vit K1 administration [38]. Although longer-chained menaquinones like MK9 showed a high circulation half-life, they are poorly absorbed because of the increase in lipophilicity [46]. These varying features among the different vitamers are also reflected in their different potencies in the biological activity, with MK7 showing the highest bioactivity followed by MK4 and Vit K1, since it leads to the same biological effects at much lower dose [30,38]. Therefore, it is important to take into consideration all these differences between the Vit K vitamers in order to choose the kind of food or commercial supplement to consume. The serum transport and distribution to target tissues of the Vit K vitamers depend on their different lipophilicity, which influences their binding to different lipoproteins. After gut absorption, both Vit K1 and Vit K2 are taken up by the liver, where Vit K1 is mainly accumulated and utilized, whereas Vit K2 menaquinones are redistributed to extrahepatic tissue, like brain (especially MK4), bone, kidney, pancreas, and vascular tissue [5,8,38,46].

### 2.4. Biological Activity

The main and well-known function of vit K is its essential role in the post-translational carboxylation of VKDPs, being a cofactor of the γ-glutamyl carboxylase (GGCX enzyme), an integral membrane protein that catalyzes the conversion of glutamic acid (Glu) residues to γ-carboxyglutamic acid (Gla) in the so-called Gla proteins [11,12,31] (Figure 1). The active form of Vit K is the reduced form hydroquinone, produced by the reduction of VK quinone to hydroquinone (VKH2) by the Vit K epoxide reductase complex subunit 1 (VKORC1) enzyme, located in the rough endoplasmic reticulum, in proximity of GGCX. The γ-carboxylation reaction is facilitated due to the presence of a homologous 18-amino-acid sequence on VKDPs, often located near the carboxylated domain, that allows the binding to the enzyme [49]. Vit KH2 acts as a cofactor in the γ-carboxylation process being converted into Vit K-2,3 epoxide, in the so-called Vit K cycle, that enables Vit K recycling for the carboxylation reactions required. As a matter of fact, VitK-2,3 epoxide (or KO) is first reduced into the quinone and subsequently to hydroquinone by VKORC1, restarting the cycle [50]. When VKORC1 is inhibited by anticoagulant drugs, like the Vit K antagonist, the conversion of Vit K quinone to hydroquinone can be ensured by the NAD(P)H-dependent oxidoreductase, an enzyme placed in the endoplasmic reticulum, also known as ferroptosis suppressor protein 1 FSP1. However, Vit K recycling is blocked because Vit K epoxide is not a substrate of this enzyme [11,35,50,51]. The γ-carboxylation of Glu residues enables the resulting Gla protein to have a higher calcium binding ability, and this is a common feature of Gla proteins. Antiferroptotic function has also been recently discovered in the reduced forms of Vit K, i.e., phylloquinone, menaquinone-4, and menadione. Previously, vitamin E was mainly known to detain this activity, yet recent studies have also shed light on Vit K vitamers. From a biochemical point of view, this inhibitory property is given by the head hydroquinone group of Vit K isoforms. The antiferroptotic activity of the reduced forms of Vit K has been observed in vivo in mouse models and in vitro in other human cell lines [52].

Ferroptosis suppressor protein 1 (FSP1), a NAD(P)H-ubiquinone reductase, efficiently reduces Vit K to its hydroquinone form, thus producing an active antioxidant and inhibitor of (phospho)lipid peroxidation. Furthermore, FSP1 is involved in the antidotal effect against warfarin, detrimental lipid peroxidation in cells, and ferroptosis [51].

### 2.5. Vitamin K-Dependent Proteins

Up to now, 17 human Gla proteins have been identified, although their number may potentially increase according to bioinformatic analyses [20,31]. These proteins are mainly involved in the blood coagulation process, bone homeostasis, and vessel calcification, and, beyond a classification according to their different biological effect, they could be classified in hepatic and extrahepatic VKDPs (Table 1). Extrahepatic Gla proteins comprise osteocalcin, Matrix Gla Protein (MGP), growth arrest-specific protein 6 (Gas6), proline-rich Gla proteins (PRGP1 and PRGP2), transmembrane Gla proteins (TMG3 and TGM4), periostin, and transthyretin. They are not involved in the blood coagulation process but are mainly related to bone and cardiovascular health, although more complex effects need to be still elucidated. Hepatic VKDPs include proteins involved in blood clotting: coagulation factors II (prothrombin), VII, IX, and X and the anticoagulant proteins C, S, and Z [11,30,53]. These could be also divided in other subgroups on the basis of the pathologic disorder related to the specific protein defect [20,54]: proteins associated only with bleeding, like FX, those associated only with thrombosis, like proteins C and S, proteins related to both bleeding and thrombosis (FII, FVII, and FIX), and proteins that are not associated with bleeding or thrombosis, like protein Z. Lastly, GGCX is a VKDP protein itself, but it does not show any homology with the other VKDPs, since it is required for other Gla protein formation so the proprotein activation of this enzyme is not needed [5,49].

Vit K is also involved in brain health by modulating different growth and cell survival factors widely expressed in the brain, e.g., Gas6 [13,14]. Gas6 is a secreted growth factor that, along with its main receptor, the TAM tyrosine kinase receptor AXL, is a crucial component in the protection of neurons and the blood–brain barrier (BBB) from inflammation and pathogen-related damage. Experimental studies suggest that suppression of the AXL-Gas6 axis in knockout mice negatively influences the progression of neuron and BBB damage following Japanese Encephalitic Virus (JEV) infection [67,75]. Gas6 may also be beneficial in regards to neurodegenerative conditions as a whole, particularly promoting the re-uptake of amyloid plaques in AD microenvironments, although this result was accompanied by a behavioral worsening in APP/PS1 mice models [76].

Vit K is also involved in the activation of the anti-thrombotic and neuroprotective protein S [77]. This protein is an important anticoagulation factor in parallel with protein C that interacts with factors Xa and Va. Although it is most known for its role in coagulation inhibition, protein S may also play a role in upkeeping brain health by contrasting ischemic events [77]. Since ischemia participates in Alzheimer’s disease (AD) pathogenesis through cerebral insults and accumulation of β-amyloid and phosphorylated tau protein, the anti-ischemic and neuroprotective properties of protein S may actively decrease the risk of AD [78,79]. Protein S has also been observed as a potential ligand for TAM receptors (namely, AXL and Mer), elucidating preventive effects on atherosclerosis [80].

Vit K is also involved in the biosynthesis of sphingolipids, essential for maintaining physiological neuro functions [23,24]. Moreover, in animal studies, diets low in Vit K result in alterations of sphingolipid profiles in the hippocampus [25,81]. Moreover, the role of Vit K, in the hydroquinone form, as an antioxidant compound and an anti-inflammatory agent is now well known [11,13]. The intracellular antioxidant activity is mediated by a VKORC1-like enzyme, a paralogue enzyme of VKORC1 [11]. Recent studies showed that Vit K inhibits ferroptosis, acting as a powerful antioxidant able to trap free radicals and therefore reducing lipid peroxidation [82].

Gla proteins involved in the coagulation cascade were the first VKDPs to be well described, and they are typically completely carboxylated, in contrast to the extrahepatic ones. These clotting factors share a common domain of 9–13 Glu residues at the *N*-terminus of the protein, also known as the Gla domain, that undergoes carboxylation to make these proteins biologically active. After the γ-glutamyl carboxylation reactions are completed on the proprotein, the prosequence is removed, and the mature protein is secreted [83,84].

### 2.6. Vit K Deficiencies

Vit K deficiency is not very common in adults and is usually associated with specific conditions, such as malabsorption disorders, antibiotics, and drug interactions, especially with anticoagulants or an extremely poor Vit K-content diet [53]. Interestingly, risk for Vit K deficiency does not increase with age; instead, infants are most at risk, since maternal milk is a scarce source of Vit K and newborn microbiota are not yet mature enough to sustain an endogenous production. The condition that arises from hypovitaminosis K in infants is a public health issue, and protocols for prophylaxis during the first weeks of life have been defined [5,85]. Excessive concentrations of Vit K are extremely rare and are to be considered when levels exceed 1000 times the daily recommended amount (RDI: 120 micrograms for adult males and 90 micrograms for adult females [86].

Vit K deficiency is clinically characterized by a bleeding tendency due to the loss of function of Vit K-dependent hepatic clotting factors. In fact, proteins related to the coagulation cascade are malfunctioning and appear to be under- or un-carboxylated, thus representing an index of Vit K insufficiency status [87]. Given the internal production of Vit K2 by the intestinal microbiota, dysbiosis may represent a cause for Vit K deficiency if dietary intake is not enough.

Furthermore, deficient Vit K levels appear to be a cause of impairment of calcium homeostasis, a so-called “calcium paradox” phenomenon, characterized by low calcium deposition in the bone and its accumulation in the vessel wall. Indeed, the un-carboxylation of MGP protein directly influences ectopic deposition of mineral matrix in the vessel wall, causing vascular calcification [58]. In particular, calcification initiates with the formation of phosphate–calcium complexes, associated with biochemical imbalance of the vascular microenvironment. Calcification of vessels is intimately associated with arterial stiffness and atherosclerotic vases, causing a deterioration in cardiac and cerebrovascular health [88]. Vascular calcification is frequently observed as a pre-existing condition in aging and several primary chronic metabolic and cardiovascular conditions, such as hypertension, diabetes mellitus, and chronic kidney disease, representing an important risk factor for cardiovascular morbidity and mortality [89,90,91,92,93].

## 3. Vitamin K in Cerebrovascular Diseases

So far, studies have shown distinct effects of Vit K forms on risk for cardiac and cerebrovascular disease [11,94] (Figure 2). Cerebrovascular diseases (CVDs) encompass a group of vascular conditions affecting the blood vessels and the blood flow specifically into brain tissues, thus causing disruption in the oxygen and nutrient supply. Stroke is one of the most common CVDs. It occurs when blood clots hinder the blood flow in an artery supplying the brain (ischemic stroke) or when a blood vessel in the brain ruptures due to structural abnormalities or other factors (hemorrhagic stroke). Depending on the stroke type, location, and extensiveness of tissue damage, impairment of neurological functions can occur, caused by local hypoxia and reduction in supply of nutrients [95].

Overall, vascular calcification can be considered an independent predictor of CVD adverse events. Accordingly, calcification of vases and arteries correlates positively with total atherosclerotic burden on the cardiovascular system [96]. Stroke and coronary artery disease are also positively correlated with coronary artery calcium scores (CACs), since individuals with CAC scores close or equal to zero have lower risks of CVD and stroke, while as CAC increases, so does the risk of these events [97,98,99].

The broad actions of Vit K in coagulation, calcium homeostasis, and vascular health have been widely described; imbalances in Vit K levels, whether due to deficiency or excess, have been observed to influence stroke risk through multiple pathways. MK-4, through its potential antioxidative and anti-inflammatory properties, has shown to be able to rescue from transient global cerebral ischemic damages in murine models. In the study by Bahram Farhadi and Masoud, cerebral stroke was generated in mice through carotid artery occlusion and was treated with MK-4 administration to study potential beneficial effects. Results indicated a general improvement in cognitive function with decreased anxiety-like behaviors and lower neurotoxicity and cell apoptosis [100].

Use of Vit K2 has been proposed as protection against vascular calcification, particularly in populations high at risk for CVDs [101]. Accordingly, in a total of 72.874 female nurses, high Vit K1 intake was found to be a marker for low cardiovascular risk but was not associated with incidence rates of total or ischemic strokes [94]. In a mice model, cerebral stroke was induced through carotid artery occlusion and was treated with MK-4 administration to study potential beneficial effects. Results indicated a general improvement in cognitive function with decreased anxiety-like behaviors and lower neurotoxicity and cell apoptosis [100].

The de-phosphorylation and under-carboxylation of MGP (dp-ucMGP) caused by Vit K deficiency have been observed to promote vascular calcification. MGP carboxylation levels can also serve as a surrogate index for monitoring of Vit K status [102,103]. In an observational study in patients with small intestinal bacterial overgrowth (SIBO), the levels of dp-ucMGP were higher with respect to the controls and were clearly associated with an increase in arterial stiffness and subclinical atherosclerosis. Surprisingly, when dietary intake of Vit K2 between the SIBO and control groups was compared, no significant difference was observed, indicating that SIBO could be a factor for Vit K2 deficiency, instead of food intake [104].

Interactions between Vit K and other vitamins and compounds can result in an increase in stroke risk. Of note, vitamin E has an antagonistic function with Vit K, since it decreases coagulating activity by deactivating clotting factors that are downstream of Vit K [105]. Also, it is hypothesized that vitamin E may compete for the upstream enzymes that activate Vit K, namely, gamma-glutamyl carboxylase (GGCX) and Vit K epoxide reductase (VKOR) [106]. Within the context of cases of intracranial hemorrhagic strokes without clear etiology, excessive vitamin E levels were correlated with hypo-prothrombinemic effects and decreased Vit K and platelet activity, suggesting hypervitaminosis E as a probable cause. Thus, in vitamin E supplementation, profiling patients based on vitamin E and Vit K levels and activity may be useful in order to prevent stroke risk and ischemic events [107].

Some studies found Vit K deficiency is associated with impaired blood clotting, due to reduced activity of clotting factors II, VII, IX, and X, which finally lead to an increased risk of hemorrhagic events [54,108]. Interestingly, studies highlighted that 82% of chronic stroke subjects were below the recommended dietary Vit K intake levels, suggesting a potential link between insufficient circulating Vit K and post-stroke complications [109].

In a population of hypertensive adults, low circulating Vit K1 and D increased the likelihood ratio of ischemic strokes [110], since Vit K-dependent MGP requires adequate but not excessive levels of Vit K to effectively inhibit arterial calcification. Recently, studies based on Mendelian randomization (MR) protocols have shown an association between Vit K1 levels and rate risk of stroke. In MR studies, genetic variants are chosen to act as proxy indicators for environmental exposure variables to infer causality. Study on data from the MEGASTROKE consortium on patients with small vessel stroke, cardioembolic stroke, and large artery stroke (LAS) revealed that genetically determined increased Vit K1 levels are associated with a parallel increase in the risk of LAS but not with other stroke subtypes [111]. A recent large-scale MR analysis using data from the European Prospective Investigation into Cancer and Nutrition study (EPIC-CVD), Coronary ARtery DIsease Genome wide Replication and Meta-analysis plus The Coronary Artery Disease (CARDIOGRAMplusC4D), and UK Biobank cohorts highlighted a positive association between increased Vit K1 levels and coronary heart disease (CHD) risk, while reporting no causal link between genetically predicted phylloquinone concentrations and CHD risk [112].

Also, hypercoagulability can be caused by increased circulating Vit K concentration [113] affecting the risk for atherosclerosis and related ischemic strokes. Furthermore, elevated Vit K levels are associated with calcification of the coronary arteries but not with vascular calcification [114,115], emphasizing the importance of proper personalized nutrition in mitigating the risk of sCVDs.

Incidentally, associating dephosphorylated and un-carboxylated matrix Gla-protein (dp-ucMGP) levels to CHD risk has yet to be done [89]. Observational studies have not established clear Vit K intake thresholds with the objective to optimize cardiovascular outcomes because of the complexity that shrouds Vit K’s role in vascular health. Indeed, observational studies generally do not support a strong link between dietary Vit K1 intake and overall stroke risk, suggesting the need for further investigation into specific subtypes like LAS [111,116].

## 4. Vitamin K in Dementia

Emerging evidence suggests an interplay between Vit K status, vascular health, and development of neurodegenerative conditions, such dementia and AD [13]. Dementia is an umbrella term used to describe a range of neurological conditions affecting the brain, causing progressive cognitive impairment. AD is the most common form and accounts for two-thirds of dementia cases; vascular dementia is the second most common, and it is associated with disease in the blood vessels in the brain, often secondary to stroke. Other forms include Frontotemporal dementia, Lewy body, and Parkinson’s disease dementia, chronic traumatic encephalopathy dementia, and others, differing for etiology and clinical features. Moreover, mixed dementia may occur, thus complicating the identification of primary cause of dementia, early diagnosis, and choosing the best therapy. The “Lancet Commission on dementia prevention, intervention, and care” has provided evidence that twelve potentially modifiable risk factors affect the development of dementia: less education, hypertension, hearing impairment, smoking, obesity, depression, physical inactivity, diabetes, low social contact, alcohol consumption, traumatic brain injury, and air pollution. Compared to the general older population, people with dementia have increased rates of cerebrovascular disease [117,118,119,120], stroke [121] Parkinson’s disease [117,119] diabetes [119,121] and others.

Hence, Vit K may play a dual role both in prevention from cardiovascular risks in subjects at risk of vascular dementia and in potential neuroprotection from AD, including global pathology, amyloid plaque and neurofibrillary tangle formation, and cognitive decline.

In dementia of vascular origin, atherosclerotic calcification can affect the brain and neuronal connections and therefore increase risk for cognitive decline and dementia [122,123,124]. Reduction in vascular calcification through adequate Vit K intake has been hypothesized as a potential strategy to preserve the cerebral blood flow, thus mitigating the risk of secondary neurodegenerative processes [13,125,126].

### Vitamin K and Alzheimer Dementia

Recent studies on vitamin K2 have shown very promising results against AD in the prevention of apoptosis, oxidative stress, and microglia activation, as well as a protective effect on cognitive functions and the inhibition of inflammation and amyloid aggregation [13].

In AD, abnormal molecular and chemical changes cause accumulation of toxic forms of Amyloid-β (Aβ), which aggregate in oligomers and fibrils up to insoluble plaques (senile plaques), as well as hyperphosphorylation of microtubule-associated tau protein and formation of the so-called neurofibrillary tangles, blocking the neuron transport system. Levels of fluid biomarkers amyloid 42 and 40, tau, and phospho tau 181 and 217 in the cerebrospinal fluid or blood reflect changes occurring in brain tissue and are helpful in routine diagnosis, although the use in blood is still challenging [103,104]. Widespread oxidative stress, glial activation, and inflammation thus exacerbate the diffusion of widespread damage to the brain, loss of connections, and cell dysfunction. Interestingly, in studies investigating alterations in proteomic expression, VKDP protein S was highlighted to be a biomarker for microglial activation in the hippocampus of AD model mice [127]. Furthermore, a number of vascular issues, including Aβ deposits in brain arteries, atherosclerosis, and vascular calcification, can damage blood vessels and reduce the flow of oxygen and nutrients to brain tissue, resulting in increased risk of mixed AD or vascular forms of dementia [128].

In transfected astroglioma C6 cells, Vit K2 treatment dose-dependently decreased the death of neural cells induced by Aβ peptides, reducing the ROS formation and inhibited the caspase-3 mediated apoptosis; the protective effect of Vit K2 was able to be abolished by administration of warfarin, indicating that the mechanism underlying the Vit K2 protection is likely against Aβ-mediated apoptosis [129].

Experimental studies on neuroblastoma SH-SY5Y cells treated with Vit K2 significantly reduced neuronal cell death, down-regulating the expression of Glycogen synthase kinase 3α/β (GSK3α/β), a serine/threonine kinase crucial in cancer, along with the levels of total tau protein, with a slight effect on secreted Aβ42 levels [130]. 

Using proteomic analysis, vitamin K-dependent protein S has shown to be a biomarker of microglia activation in the hippocampus of 5XFAD mice, which carry mutations in APP and PSEN1 genes; further, serum level of protein S increased as the disease progressed. Similarly, in AD patients, serum protein S increased, showing a close correlation with AD neuroimaging markers [127]. Other omics studies found correlations with other VKDPs, such as transthyretin (TTR) [131].

Vit K concentration offers interesting insights to consider when studying cognitive decline in aging subjects and elder community dwellers. A recent study investigated the association between serum phylloquinone concentrations, a biomarker of Vit K status, and cognitive performance, using data from the Québec Longitudinal Study on Nutrition and Successful Aging (NuAge) [132]. The study involved 320 cognitively healthy participants aged 70–85 years and found significant positive association between increased phylloquinone serum levels and enhanced performance in verbal episodic memory tasks and memory consolidation but not in executing tasks, non-episodic memory, and processing speed. Learning and delayed recall trials showed a net improvement in subjects with higher phylloquinone serum levels, suggesting that Vit K may help with stabilizing memories. A study investigated Vit K levels in brain tissues in correlation with cognitive function and neurodegeneration, comparing ante-mortem cognitive performances with post-mortem cerebral concentrations of Vit K vitamers and neuropathological outcomes. The results confirmed the association between high phylloquinone plasma levels and slowed cognitive decline but also showed that increased levels of brain menaquinone positively correlated with brain health. In particular, decreased AD-related neuropathology was observed in post-mortem analysis in patients with high MK-4 brain levels along with fewer neurofibrillary tangles and a lower chance of Lewy body presence [133]. During the ELDERMET studies, which included a cohort comprising 156 older adults, the link between cognitive function, Vit K status, and inflammation was examined [134]. Results highlighted an increased level of dietary and serum phylloquinone in patients with increased cognitive function. Concurrently, inflammatory markers such as IL-6 were elevated in subjects with poorer cognitive function. Specifically, dietary and serum phylloquinone levels were significantly lower in the group with severe cognitive decline (i.e., Mini-Mental State Examination, MMSE, score lower than 15). These findings suggest that Vit K status could be useful a useful parameter in strategies of prevention for cognitive decline [134]. When comparing the dietary intake of vitamin K between patients with early-stage AD and cognitively normal controls, it was found that the former consumed significantly less vitamin K than the latter. Green vegetables, which are the main source of vitamin K, accounted for 33% of the patients’ total intake, while the controls consumed 49%. This suggests that Vit K intake could either serve as a risk factor for AD or accelerate its progression, although green vegetables contain other nutrients, such as folate and carotenoids, which may also be linked to cognitive health [135].

Further studies in AD patients demonstrated that in apolipoprotein E4 (APOE4) carriers, the strongest genetic risk factor for AD [136], lower blood concentrations of Vit K, were observed when compared to other APOE genotype carriers [137,138]. In APOE^-/-^ murine models with Toll-like receptor (TLR) activity, mice fed with a high fat diet and supplemented with Vit K2 showed significantly lower levels of aortic atherosclerosis and calcification [139], suggesting the effectiveness of Vit K in consolidating cardiovascular health in conditions where APOE variants and lifestyle choices are adverse. ApoE4 plays a significant role in lipoprotein-related uptake of Vit K in osteoblasts [73,74,136]. Lower Vit K levels in ApoE4 carriers can be explained by the increased lipid uptake capabilities exhibited by the E4 variant. Greater lipid affinity by ApoE4 has been associated with a point mutation in the aminoacidic sequence of the protein that renders it more structurally flexible [136].

Finally, vitamin K and other vitamins, namely, A, D, and E, are reported to influence further mechanisms involved in AD pathogenesis, e.g., Aβ-aggregation, Aβ-induced neurotoxicity, oxidative stress, and inflammatory processes, contributing to decreasing Aβ plaques accumulation in AD cases and inhibition of plaque formation [140]. This could be amazing for the treatment of AD and other proteinopathies. Notwithstanding, further studies are needed to effectively evaluate the rate and efficacy of aggregate removal and the effect on amyloid accumulation.

Clinical trials of new drugs for AD are still ongoing, but despite great efforts, no drug has yet been identified that can stop the disease. Recently, Lecanemab, an antibody that targets amyloid proteins, has received approval by the United States Food and Drug Administration (USFDA) and the European Medicines Agency (EMA), although numerous restrictions and evaluation on safety and efficacy are still under consideration [141]. Indeed, limitations for use of Lecanemab include patients using anticoagulant agents for conditions such as atrial fibrillation (AF), deep vein thrombosis, or pulmonary embolism or subjects with hypercoagulable state or cerebral hemorrhage, among others [141]. Then, defining alternative strategies for the use of natural compounds for AD prevention is a challenge to decrease the incidence and the impact of the impairment and disability on patients.

Supplementing antioxidant compounds and enzymatic compounds with proteolytic activities may ameliorate the brain health and stimulate the amyloid clearance [13,142,143,144]. Vit K has been considered as a prospective nutritional component [145], given the ability of Vit K to efficiently neutralize reactive oxygen species (ROS) (Figure 2). However, supplementation studies suggest Vit K can improve status but not normalize it completely [146]. Beyond its antioxidant properties, Vit K2 also exhibits anti-apoptotic effects [147], which may finally result in neuron protection from cell death induced by Aβ [124]. In experimental studies on cellular models, Vit K2 also emerges as a potential neuroprotective agent for counteracting ROS production and tauopathy [148], whereas in rats, low Vit K intake is associated with development of cognitive impairment [149].

## 5. Vitamin K and Aging

Diseases and conditions that are mainly related to aging are on the rise in industrialized countries due to the demographic changes in the last decades [150]. Lifestyle choices and diet influence the incidence and severity of a number of diseases related to aging, ranging from osteoporosis to dementia. Use of Vit K may represent a tool for both prevention of neurodegenerative processes and protection against age-related conditions.

Vit K is increasingly recognized as a vital nutrient for bone health through activation of VKDPs like osteocalcin, essential for calcium provision to the bone matrix and bone mineralization, thus preventing conditions like osteoporosis [125]. MK-4 and MK-7 isoforms have demonstrated interesting benefits by improving bone mineral density (BMD) and preventing bone fractures [151]. This is especially crucial in postmenopausal women and individuals with osteoporosis, where higher doses of vitamin K2 have been linked to greater improvements in bone strength and a lower incidence of fractures. Moreover, regular intake of vitamin K-rich foods like green leafy vegetables and dried plums has been associated with reduced levels of un-carboxylated osteocalcin (ucOC), a marker of vitamin K deficiency linked to poor bone quality and increased fracture risk [152].

Aging has an impact on the composition of intestinal microbiota, with implications on gut–brain axis dysregulation and occurrence of several neurodegenerative and cerebrovascular conditions. In particular, Vit K production by human gut bacteria may be altered by factors such as age, sex, and lifestyle choices [5]. During age-related dysbiosis, a state characterized by an imbalance between beneficial and harmful bacterial populations, chronic inflammation, oxidative stress, and vascular dysfunction may become exacerbated [153]. A metagenomic study revealed the association between MK biosynthesis genes and clinical parameters, including cognitive decline. By sequencing the microbial metagenome of 74 fecal samples, four clusters with similar concentrations of predicted MK production capacity have been identified, with only three MKs being similar across all clusters (MK 4, 6, and 7], and certain MK isoforms were associated with specific clusters, particularly three MKs that had a positive association with MMSE (MK 6, 12, and 13). In particular, cluster 1 MK biosynthesis genes were associated with poorer health, including the lowest MMSE and Barthel score, higher TNF-α, lower microbial diversity, and a higher proportion of subjects residing in long-term care. In a murine model of dysbiosis induced by ampicillin, a cognitive decline in certain behavioral aspects was observed. K2 administration reversed the gut dysbiosis-associated cognitive decline, further reducing the levels of acetylcholinesterase and oxidative stress markers, suggesting a delicate interplay between gut microbiota, Vit K production, and cognitive status [154]. Indeed, eating patterns can be a strategy for regulating Vit K and other metabolites that promote gut microbiome health [155]. 

The increased intestinal permeability caused by age-related dysbiosis alongside the disruption of the BBB exposes neurons to bacterial endotoxins from the gut microbiota, further worsening oxidative stress in neurodegenerative conditions [156]. Thus, gut–brain axis dysregulation may affect stroke outcome and recovery, since vascular inflammation can be influenced by gut dysbiosis through modulation of oxidative stress, apoptosis, and inflammation [109,157].

## 6. Use of Vitamin K Antagonist

In frail patients suffering from vascular disorders, atherosclerosis, chronic conditions, or a history of stroke, constant monitoring of blood coagulation would be recommended.

Vit K antagonists (VKAs) are one class of compounds whose anti-Vit K effects are exploited to prevent thrombotic events and act as ‘’blood thinners’’. These anticoagulants act by antagonizing Vit K to reduce clotting, which can affect the occurrence of bleeding and hemorrhagic events [158]. VKAs target both post-translational carboxylation of coagulation factors by Vit K and synthesis of extrahepatic Vit K-dependent proteins [159]. VKA are effective as anticoagulants but may conceal potentially dangerous side effects regarding associated coagulation risks.

Warfarin, one of the most widely used VKAs, has been a cornerstone in anticoagulant therapy for decades [160]. It functions by inhibiting the synthesis of Vit K-dependent clotting factors, thereby reducing the blood’s ability to clot. This mechanism is effective in lowering the risk of stroke in patients with atrial fibrillation [161]. By influencing Vit K availability, warfarin also influences MGP activity, and its use has been linked to vascular calcification [162] due to under-carboxylation of MGPs [159]. Warfarin use in stroke prevention requires regular monitoring to ensure therapeutic effectiveness and minimize bleeding risks. Maintaining an international normalized ratio (INR) of 2.0 or greater has been shown to not only reduce the frequency of ischemic stroke but also its severity and mortality [163].

In recent years, direct oral anticoagulants (DOACs) have emerged as alternatives to Vit K antagonists, thanks to the beneficial effect in mitigating side effects and hemorrhaging risks. These include factor Xa inhibitors (such as apixaban and edoxaban) and direct thrombin inhibitors (such as dabigatran). DOACs offer several advantages over warfarin, including fixed dosing and fewer dietary restrictions, and above all, they do not require routine INR monitoring. Furthermore, DOACs are preferable to warfarin in treating stroke in patients with AF and chronic kidney diseases [164] since they have demonstrated to be at least as effective as warfarin in preventing stroke and systemic embolic events in patients with AF, with comparable or even reduced risks of major bleeding [165].

## 7. Conclusions: Vitamin K, Prevention, and Therapeutic Approaches

Here, we discuss the state of the art on knowledge on vitamin K in neurological diseases associated with coagulation defects and a possible application in the prevention of neurodegenerative conditions. Compelling clinical evidence in patients along with studies in experimental models clearly support the beneficial dual effects of Vit K in prevention of cerebrovascular and degenerative conditions by protecting the cerebral blood flow and thus acting in a plethora of neuropathological conditions. In stroke, Vit K seems to have a protective role, preserving vessels and walls from calcification; furthermore, in the elderly population, the reduction in Vit K increases the occurrence of cognitive impairment, whereas Vit K seems to be a neuroprotective agent for cognitive decline.

Like Janus Bifrons, the Roman god depicted with two faces looking simultaneously in opposite directions, past and future, vitamin K may also have a dual role: in stroke, for prevention in patients with AF and vascular conditions and in AD, as support in protecting against the neuropathological mechanisms of the disease.

Another interesting field could be to investigate the relationship between vitamin K and markers of neuronal damage and degeneration to explore correlations between Vit K status with biomarkers changes. In a population of healthy subjects, Vit K and serum neurofilament light (NfL) concentration, a marker of axonal damage, followed a non-linear negative dose-response [166]. Research on neuronal biomarkers is still unfolding, yet future efforts should also be oriented towards studying the trend of these biomarkers in response to Vit K status.

Regarding the use of K supplements, Vit K is safe and has a sustainable cost, representing a significant impact on public health; excess levels are not dangerous for health, since taking high doses of Vit K is not associated with an increased thrombosis tendency [167].

Although further studies are needed to establish the optimal concentration of a combined Vit K supplement, their use has shown to be safe for health. In fact, high levels of Vit K1, MK4, or MK7 have not been documented to cause toxicity or adverse health effects. No hypercoagulable state was observed in individuals after MK7 supplementation [168,169]. Moreover, specific cases of extremely high levels of Vit K intake have also been reported without adverse effects [170,171].

However, current knowledge of the different types of Vit K vitamers, particularly their specific biological activity, requires careful consideration of their potential clinical and biological significance and effect, depending on properties and specific targets. Both Vit K1 and Vit K2 may play a role in the pathogenesis and progression of many diseases due to their effect on multiple pathways, from vascular calcification, inflammation, and the potential role on amyloid clearance; however, the Vit K2 vitamer seems to have a better efficacy due to a higher bioavailability and half-life compared to other Vit K vitamers.

Finally, the majority of available clinical studies on Vit K1 still concern health effects, while Vit K2 has shown a predominant function in extrahepatic tissues, for example, a protective role in the vascular system by reducing the risk of cardiovascular disease and cognitive impairment and suppressing inflammation.

A limitation of this narrative review is the lack of a qualitative and quantitative methods to analyze all the available evidence on the clinical efficacy of vitamin K in an attempt to understand the real impact on health and prevention. A systematic review is needed to answer the many questions, such as defining the differences in the doses used or the impact on the prevention of stroke or cognitive impairment.

On the other hand, anticoagulants targeting Vit K, such as warfarin, are used to prevent harmful blood clots that can block blood flow to the brain or heart by decreasing or delaying the clotting ability of Vit K. In these cases, suddenly increasing or decreasing Vit K intake can interfere with the effects of these drugs, with potentially dangerous effects. Careful monitoring of Vit K intake and levels is crucial to preventing severe effects and assessing the rate of other conditions, such as calcification and clotting.

Vit K holds significant promise as a nutraceutical intervention mainly for mitigating risks associated with cardiovascular diseases, aiding against vascular calcification and further promoting antioxidant and anti-amyloidogenic properties. While existing studies highlight its potential, further clinical trials are necessary to refine dosing strategies and fully understand its therapeutic scope.

## Figures and Tables

**Table 1:** Localizations and functions of VKDPs.

Localization	Proteins	Functions	References
Hepatic	Coagulation factors		
	FII (Prothrombin), FVII, FIX, FX	Procoagulant	[6]
	Anticoagulation factors		
	Protein C	Anticoagulant, anti-inflammatory, anti-thrombotic	[6,55]
	Protein S	Anticoagulant, regulation of hemostasis, bone homeostasis	[56,57]
	Protein Z	Anticoagulant, anti-thrombotic	[6]
Extrahepatic	Matrix Gla Protein (MGP)	Vascular calcification, bone formation	[58,59,60]
	Proline-rich Gla proteins (PRGP1/PRGP2)	Signal transduction	[61,62]
	Gla-rich protein (GRP)	Repression of osteogenic differentiation	[63,64]
	Transmembrane Gla proteins (TMG3/TMG4)	Signal transduction	[65]
	Growth arrest-specific protein 6 (Gas6)	Signal transduction, apoptosis, anti-inflammatory, platelet activation, thrombus stabilization, microglial and astrocyte activation	[30,66,67,68,69]
	Osteocalcin	Bone formation, osteoporosis	[70]
	Periostin	Angiogenesis, bone matrix reparation	[71]
	Transthyretin	Hormone transportation (plasma and cerebrospinal fluid)	[72]
	Apolipoprotein E (ApoE)	Lipid transport	[73,74]
Endoplasmic Reticulum/Golgi Apparatus	Gamma-glutamyl carboxylase (GGCX)	γ-carboxylation	[49]

## Data Availability

No new data were created or analyzed in this study. Data sharing is not applicable to this article.
